# Investigating Outcomes of Gamete Donation in Assisted Reproductive Technology: A Retrospective Study

**DOI:** 10.5935/1518-0557.20250056

**Published:** 2026

**Authors:** Sofia Soares, Carla Leal, Márcia Barreiro, Emídio Vale-Fernandes

**Affiliations:** 1 ICBAS - School of Medicine and Biomedical Sciences, UMIB - Unit for Multidisciplinary Research in Biomedicine, University of Porto, Porto, Portugal; 2 Centro de Procriação Medicamente Assistida / Banco Público de Gâmetas, Centro Materno-Infantil do Norte Dr. Albino Aroso (CMIN), Centro Hospitalar Universitário de Santo António (CHUdSA), Unidade Local de Saúde de Santo António (ULSSA), Porto, Portugal; 3 ITR - Laboratory for Integrative and Translational Research in Population Health, University of Porto, Porto, Portugal

**Keywords:** gamete donation, oocyte donation, sperm donation, assisted reproductive technology, infertility, obstetric outcomes

## Abstract

**Objective:**

This study aims to evaluate and characterize Assisted Reproductive Technology (ART) cycles, as well as the associated obstetric and neonatal outcomes, in pregnancies resulting from gamete donation at the Public Gamete Bank of the Centro Materno Infantil do Norte Dr. Albino Aroso (CMIN), Unidade Local de Saúde de Santo António (ULSSA), in Porto, Portugal.

**Methods:**

We conducted a retrospective analysis of 238 gamete donation cycles from 2011 to 2021. Collected variables included recipient age, duration and cause of infertility, number of attempts, ART techniques employed, embryo transfer details, beta-human chorionic gonadotropin (β-hCG) values, live birth rates, gestational age, mode of delivery, and birth weight. Statistical analyses were performed using IBM SPSS Statistics 27, with a significance threshold set at *p*<0.05.

**Results:**

Among sperm donation cases, 29.4% resulted in preterm deliveries, with 24.3% of pregnancies being non-ongoing and 47.7% leading to cesarean sections. Low birth weight was observed in 29.3% of singletons and 69% of twins. Multiple gestations occurred in 24.5% of cases, with a significant increase when two embryos were transferred (*p*=0.027). In oocyte donation situations, 40% of pregnancies resulted in preterm deliveries and 43.4% experienced low birth weight. Multiple pregnancies increased the risk of preterm delivery and low birth weight.

**Conclusions:**

The high incidence of multiple gestations underscores the need for single embryo transfer, particularly for older oocyte recipients. It is crucial for couples to be educated about the associated risks and benefits of this approach, alongside the implementation of comprehensive maternal and fetal monitoring throughout the reproductive process.

## INTRODUCTION

According to the World Health Organization (WHO), infertility is defined as the inability to conceive after 12 months or more of regular, unprotected sexual intercourse, or after 6 months for women over the age of 35 (World Health Organization (WHO), 2018). It impacts around 17.5% of the adult population - roughly 1 in 6 worldwide (World Health Organization (WHO), 2021). Gamete donation is employed in Assisted Reproductive Technology (ART) when infertility causes preclude the use of the individuals’ own gametes or in the presence of genetic, infectious, or other anomalies (Direção Geral de Saúde, 2011). Since 2017, in Portugal, all women have been permitted to access ART techniques regardless of their marital status, sexual orientation, or infertility diagnosis (Assembleia da República, 2017).

Gamete donation was legalized in Portugal in 2006 (Assembleia da República, 2006) and has become an increasingly common treatment option for women of all ages, not only those of advanced reproductive age worldwide (Jeve *et al.*, 2016). The indications for oocyte donation include Premature Ovarian Insufficiency (POI), the inability to conceive with own oocytes due to genetic or other reasons, and repeated unsuccessful cycles of ART. The indications for ART using donor sperm include azoospermia, genetic anomalies in heterosexual couples and treatment for single women, female same-sex couples or transgender individuals.

Embryo transfer is conducted based on the embryonic development, morphology, and cell number. The number of embryos to be transferred is a current topic of debate and has evolved with the implementation of elective Single Embryo Transfer (eSET) aimed at reducing the incidence of multiple gestations. Remaining embryos may be cryopreserved. ART techniques are increasingly utilized, and the associated risks have decreased over time; however, complications remain including an increased rate of caesarean delivery, preterm birth, multiple gestation and low birth weight. As techniques continue to evolve and the use of gamete donation rises, there is ongoing research into the possibility that gamete donation may constitute an independent risk factor for these complications. Additionally, various factors may be potentially associated with improved or poorer obstetric and neonatal outcomes.

This process inherently imposes a significant emotional burden on the couple/woman, emphasizing the importance of continued research to ensure optimal counseling. This investigation aimed to study the different cycles of ART and the obstetric outcomes of pregnancies resulting from gamete donation at the Public Gamete Bank (*Banco Público de Gâmetas*, in Portuguese) of Centro Materno Infantil do Norte Dr. Albino Aroso (CMIN), Unidade Local de Saúde de Santo António (ULSSA).

## MATERIAL AND METHODS

### Study Design and Population

This retrospective cohort study examined all pregnancies resulting from gamete donation at the Public Gamete Bank of Centro Materno Infantil do Norte Dr. Albino Aroso (CMIN), Unidade Local de Saúde de Santo António (ULSSA), from 2011 to 2021. Eligible cases included all ART cycles utilizing donated gametes that led to a confirmed pregnancy diagnosis via beta-human chorionic gonadotropin (β-hCG) measurement, irrespective of subsequent pregnancy outcomes. Exclusion criteria encompassed cases with incomplete data, including missing obstetric outcomes and lack of follow-up. Consequently, 242 procedures were initially considered; however, 4 were excluded due to incomplete data, yielding a final cohort of 238 ART cycles.

### Subgroup Classification

The cohort was stratified into two distinct subgroups based on donor type: male gamete donation encompassed 184 cycles (77.3%), while female gamete donation accounted for 54 cycles (22.7%). Within these subgroups, various clinical parameters were evaluated, including recipient age, infertility duration, infertility type and etiology, cycle attempt count, applied ART techniques, procedure date, β-hCG concentrations, and the count of embryos demonstrating cardiac activity via ultrasound.

### Outcome Measures

**Figure 1:**
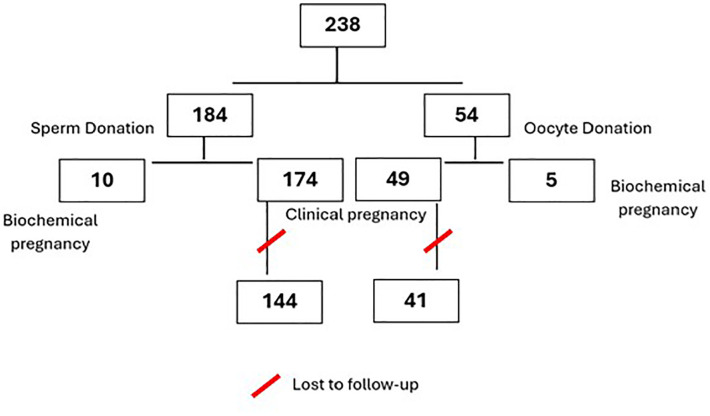
Overview of Study Sample Size and Participant Follow-Up Loss.

Several outcome metrics were assessed, including live birth rates, gestational age at delivery, mode of delivery, birth weight, and where applicable, details regarding embryo transfer day, number of embryos transferred, and the type of transfer (fresh vs. cryopreserved embryos). Analysis of these variables was restricted to a subset of the population due to exclusion based on loss to follow-up (Figure 1).

### Infertility Definitions and Classifications

Infertility duration was quantified in months from the recipient’s initiation of the conception process. Infertility was categorized as primary or secondary, differentiating between individuals with no prior pregnancy *versus* those with a history of pregnancy. The etiologies of infertility for sperm donation included azoospermia, single-parent arrangements, transgender recipient factors, and combined male and female factors (with female factors comprising conditions such as endometriosis, polycystic ovary syndrome, other anovulatory disorders, uterine anomalies, and tubal obstruction, in addition to genetic considerations like Klinefelter syndrome). In contrast, factors leading oocyte donation included oocyte quality issues (e.g., POI), genetic conditions (e.g., gonadal dysgenesis, maternal mitochondrial diseases, gonadoblastoma), and the history of unsuccessful ART cycles.

### ART Techniques and Measurements

ART attempts were defined with the first attempt representing the initial ART cycle conducted. The ART methodologies for sperm donation included intrauterine insemination (IUI) as the primary technique, alongside second-line techniques such as *in vitro* fertilization (IVF) and intracytoplasmic sperm injection (ICSI). Both IVF and ICSI were similarly employed in oocyte donation cases. β-hCG levels were quantified in mIU (international units)/mL approximately 14-16 days post-ART intervention, serving as a marker to differentiate biochemical pregnancies (where no gestacional sac is detected on ultrasound 2 weeks later) from clinical pregnancies. Clinical pregnancies could be further classified as singleton or multiple pregnancies based on the number of embryos visualized. A miscarriage is characterized by the cessation of embryonic cardiac activity, whereas an ectopic pregnancy is defined as a pregnancy that occurs outside the intrauterine cavity.

The live birth count enabled the determination of successful ongoing pregnancies (defined by the birth of one or more live newborns) *versus* unsuccessful pregnancies (characterized by miscarriage or other adverse outcomes). Gestational age was measured in weeks, categorizing births as preterm (before 37 weeks) or term (at or after this threshold).

### Mode of Delivery and Newborn Metrics

Delivery modalities were delineated as spontaneous vaginal delivery (eutocic), assisted vaginal delivery (dystocic, employing instruments such as forceps or vacuum extraction), or cesarean section (dystocic). Newborn weight was recorded in gram, with low birth weight being designated as under 2500 g. Weight assessments were conducted for the first newborn (singleton context) or all newborns (in multiple gestations).

### Embryo Transfer Classification

Regarding the day of transfer for second-line techniques, embryos were classified as cleavage-stage (transferred on days 2-3) or blastocyst-stage (transferred on days 5-6).

### Ethics and Data Analysis

Approval for the study was obtained from the Ethics Committee of ULSSA/School of Medicine and Biomedical Sciences (ICBAS) [study reference: 2021.329(274-DEFI/282-CE)]. Data were analyzed utilizing IBM SPSS Statistics 27. Descriptive statistics were presented with categorical variables summarized as frequency/percentage, while continuous variables were expressed as mean and/or median. Statistical comparisons employed appropriate methodologies, including independent samples t-test, ANOVA, Chi-square test, and Fisher’s exact test, with a significance threshold set at *p*<0.05.

## RESULTS

### Sperm Donation

IVF and ICSI were categorized as second-line techniques, representing 61.4% of the total cases, while IUI was classified as a first-line technique (Table 1). The average age of female participants was 32.89 years (± 3.8). In the first-line technique group, the mean age was 32.03 years (± 3.9), whereas in the IVF/ICSI group, it was 33.43 years (± 3.7). A higher reliance on second-line techniques was observed in women over 37 years (*p*=0.063).

**Table 1:** Rate of development of transferred embryos.

	Sperm Donation (N=184)	Oocyte Donation (N=54)
**Age (years)** **Min** **Max** **Mean**	23 41 32.89	23 40 35.48
**Infertility** **Primary** **Secondary**	91.8% 8.2%	90.7% 9.3%
**Time Infertility (months)** **Min** **Max** **Mean**	4 412 61.9	24 228 79.09
**Attempts** **First** **Second** **Third**	65.8% 25.5% 8.7%	88.9% 7.4% 3.7%
**Causes** **Azoospermia** **Male + Female** **Genetic** **Monoparental** **Transsexual** **Oocyte Factor** **Previous unsuccessful cycles**	79.9% 14.1% 3.8% 1.1% 1.1% - -	- 13% 7.4% - - 75.9% 3.7%
**Techniques** **IUI** **IVF** **ICSI**	38.6% 55.4% 6%	- 44.4% 55.6%
**β-hCG** **Min** **Max** **Mean**	5.3 11669 601.7	10 7937 1000
**Number of Embryos with cardiac activity** **0** **1** **2** **3** **4**	5.4% 70.1% 22.8% 1.1% 0.5%	9.3% 66.7% 24.1% - -

β-hCG: beta-human chorionic gonadotropin; ICSI: intracytoplasmic sperm injection; IUI: intrauterine insemination; IVF: *in vitro* fertilization.

Primary infertility accounted for 91.8% of cases (Table 1). First-line techniques were utilized in 42% of primary infertility cases, whereas no first-line techniques were employed in instances of secondary infertility (100% utilized IVF/ICSI), yielding a statistically significant difference (*p*=0.001). The mean duration of infertility in the study population was 61.9 months (± 41.6) (Table 1). A significant association was observed between ART techniques and the duration of infertility; first-line techniques were associated with a shorter duration of infertility averaging 44.41 months (± 23.5), while second-line techniques related to a longer duration, averaging 72.71 months (± 46.6), demonstrating a statistically significant difference (*p*<0.001).

In terms of the number of attempts, 65.8% of cases represented the first attempt, 25.5% the second attempt, and 8.7% the third attempt (Table 1). There was no statistically significant association between the number of attempts and ongoing pregnancy outcomes or complications such as preterm birth, low birth weight, or mode of delivery (*p*=0.792).

Azoospermia accounted for 79.9% of cases, with 56.5% treated with second-line techniques (Table 1). Notably, a majority (90.1%) of the IUI cases were attributed to azoospermia. In cases of monoparental cycles, 100% utilized first-line techniques, whereas in cases with both male and female factors, 96.2% employed IVF/ICSI. A similar distribution was observed regarding genetic and transgender-related infertility causes.

The mean β-hCG level was 601.7 mIU/mL (± 1423.9) (Table 1). The mean β-hCG was higher in clinical pregnancies (627.8) compared to biochemical pregnancies (145.7), with 80% of biochemical pregnancies presenting with β-hCG values less than 100 (*p*=0.215). Although not statistically significant (*p*=0.082), the average β-hCG level in ongoing pregnancies (619) was greater than that in non-ongoing pregnancies (270.2), with 77.2% of non-ongoing pregnancies occurring with β-hCG values <300.

The median β-hCG values were recorded as follows: 72.3 mIU/mL for biochemical pregnancies, 189 for singleton pregnancies, and 382 for multiple pregnancies, with a statistically significant difference revealed by the Mann-Whitney test (*p*<0.001). No significant relationship was identified between β-hCG levels and gestational age, although the mean β-hCG level was higher in cases of preterm birth. The day of transfer did not reveal any significant associations, although higher β-hCG values were observed when embryos were transferred on days 5-6 compared to days 2-3, as well as in the context of two-embryo transfers (*p*=0.836).

Among the clinical pregnancies analyzed, 94.6% were classified as such, with 70.1% being singleton pregnancies and 24.5% multiple pregnancies (Table 1). Notably, second-line techniques were associated with a higher percentage of multiple pregnancies (29.2%) compared to first-line techniques (16.9%), although this difference was not statistically significant (*p*=0.118).

Out of 174 clinical pregnancies, 30 were lost to follow-up, resulting in 144 cases available for analysis (Figure 1). Among these, 24.3% were classified as non-ongoing pregnancies, while 75.7% were ongoing pregnancies (Table 2). A statistically significant relationship was observed between maternal age and embryo evolution/live birth outcomes (*p*<0.001). Among women older than 37 years, 71.4% of cases were non-ongoing pregnancies, in contrast to approximately 80% of cases resulting in successful pregnancies in women younger than 37 years.

**Table 2:** Obstetric and Neonatal Outcomes by donor type: Sperm Donation vs. Oocyte Donation.

	Sperm Donation	Oocyte Donation
**Number of alive newborn** **0** **1** **>1**	n=144 24.3% 55.6% 20.1%	n=41 26.8% 53.7% 19.5%
**Delivery** **Eutocic** **Vaginal Dystocic** **Caesarean**	n=109 35.8% 16.5% 47.7%	n=30 30% 13.3% 56.7%
**Gestational age** **Min - Max (weeks)** **Mean (weeks)** **Term** **Preterm**	n=109 26-41 37.13 70.6% 29.4%	n=30 28-41 37 60% 40%
**Weight** **Number of alive newborn: 1** **Mean (grams)** **Low birth weight** **Number of alive newborn: 2** **Mean (grams)** **Low birth weight** **Number of alive newborn: 3** **Mean (grams)**	n=109 2884 29.3% n=29 2194 69% n=1 1250	n=30 2108 43.4% n=8 2108 62.5% - -

Although a definitive relationship cannot be established, higher percentages of multiple live births were observed in the IVF/ICSI group (22.3%) compared to the IUI group (16%), with ongoing pregnancy rates of 74.5% for IVF/ICSI and 78% for IUI being similar. Cesarean delivery was the most prevalent mode of birth (Table 2), with IVF/ICSI having a higher rate of cesarean deliveries (55.7%) compared to IUI (33.3%). Notably, 75% of cesarean deliveries were associated with IVF/ICSI (*p*=0.072).

The majority of pregnancies resulted in term deliveries, with 29.4% classified as preterm (Table 2). The type of conception, maternal age, and duration of infertility did not appear to influence the occurrence of preterm births. A statistically significant association was found between multiple *versus* singleton pregnancies and gestational duration (*p*<0.001), whereby 65.6% of preterm births were attributed to multiple gestations, and the majority of multiple gestations (63.6%) resulted in preterm delivery.

Descriptive analysis of newborn weight, as presented in Table 2, indicated that 23.9% of firstborns and 69% of secondborns weighed less than 2500 grams. In multiple gestations, the mean weight of the first newborn was 2340.76 grams, while singleton gestations had a mean weight of 3121.09 grams. No significant relationships were identified between newborn weight and the ART technique utilized, as well as with maternal age, day of transfer, β-hCG levels, and type of embryo transfer.

Overall, with respect to the day of transfer, embryos were predominantly transferred at the cleavage stage rather than at the blastocyst stage (Table 3). No significant differences were observed in the number of embryos visualized on ultrasound (singleton *versus* multiple gestation) between cleavage-stage and blastocyst-stage transfers. Similarly, no statistically significant relationship was found regarding embryo evolution; on days 2-3, 28.8% of pregnancies were classified as non-ongoing pregnancies, compared to 19.2% for transfers on days 5-6 (*p*=0.347). Rates of preterm birth were 29.8% for cleavage-stage embryos and 33.3% for blastocysts (*p*=0.251).

**Table 3:** Characteristics of Embryo Transfer Procedures by donor type: Sperm Donation vs. Oocyte Donation.

	Sperm Donation (N=113)	Oocyte Donation (N=54)
**Day of transfer** **2/3** **5/6**	70.3% 29.7%	90.7% 9.3%
**Number of embryos transferred** **1** **2**	15.9% 84.1%	33.3% 66.7%
**Type of transfer** **Fresh (%)** **Age (mean, years)** **Cryopreserved (%)** **Age (mean, years)**	84.1% 32.97 15.9% 35.89	16.7% 35.16 83.3% 37.11

**Figure 2:**
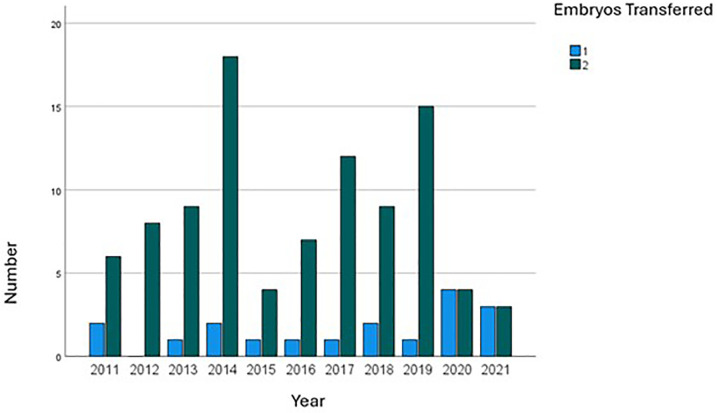
Trends in Embryo Transfer numbers over time in Sperm Donation Cycles.

In terms of the number of embryos transferred, two embryos were transferred in 84.1% of cases, constituting the majority across all age categories (Table 3). Over time, despite some fluctuations, the predominant practice remained the transfer of two embryos, although there has been a trend towards increasing eSET in recent years (Figure 2). When a single embryo was transferred, 5.6% of pregnancies were classified as biochemical, while 94.4% were clinical and singleton. In two-embryo transfers, 6.3% of pregnancies corresponded to biochemical pregnancies, 58.9% exhibited one embryo on ultrasound, 33.7% displayed two embryos, and one instance involved three embryos, resulting in 34.8% of cases classified as multiple pregnancies (*p*=0.027). The rate of ongoing pregnancies was higher in two-embryo transfers (77.5%) compared to single embryo transfers (57.1%) (*p*=0.107).

A total of 95 fresh ART cycles and 18 cycles involving the transfer of cryopreserved embryos (frozen embryo transfer - FET) were conducted. The mean maternal age was significantly higher in the FET group (35.89 years) compared to the fresh cycles group (32.97 years) (*p*=0.002) (Table 3). No statistically significant differences were noted in the number of embryos observed on ultrasound between FET and fresh cycles (*p*=0.547). The FET group yielded an ongoing pregnancy rate of 71.4%, while fresh cycles demonstrated a 75% ongoing pregnancy rate. In the context of FET cycles, the rate of preterm births was 40%, which was higher than that observed in fresh cycles (28.3%) (*p=*0.456). No significant relationships were established concerning the mode of delivery (*p*=0.611).

### Oocyte Donation

A total of fifty-four reproductive procedures were conducted utilizing both IVF and ICSI techniques (Table 1). The mean age of female participants was 35.48 years (± 3.4). No statistically significant correlation was identified between maternal age and the ART employed (*p*=0.711).

The maternal Body Mass Index (BMI) was assessed, yielding a mean value of 25.6 kg/m^2^. Among the participants, 53.7% were classified as having a normal BMI, 22.2% were classified as overweight, 16.7% had grade 1 obesity, and 7.3% had grade 2 obesity. There was no significant association between BMI and pregnancy outcomes.

The predominant etiology of infertility among the participants was primary infertility (Table 1). The distribution of infertility types across the different techniques was approximately consistent, with no statistically significant correlation identified (*p*=0.462). The mean duration of infertility recorded was 79.09 months (± 40), with ICSI demonstrating a higher mean duration (86.5 months) in comparison to IVF (69.83 months), although this difference was not statistically significant (*p*=0.747).

Regarding the number of attempts at conception, 88.9% of cases represented the first attempt (Table 1). No significant relationship was found between the number of attempts and embryo evolution outcomes (*p*=0.435).

In terms of infertility causes, oocyte factor was the most prevalent, accounting for 75.9% of cases (Table 1). Despite a limited sample size, it was observed that when oocyte factor was the etiology, the distribution was consistent with global percentages for IVF/ICSI. In cases representing male and female factors, 100% of the procedures utilized ICSI, while IVF was employed in all cases with previous unsuccessful cycles. Additionally, among cases attributed to genetic causes, 75% utilized IVF while 25% utilized ICSI.

The mean β-hCG level was 1000 mIU/mL (± 1622) (Table 1). While not statistically significant (*p*=0.198), the mean β-hCG levels were observed to be 107.5 for biochemical pregnancies and 1093 for clinically confirmed pregnancies, with biochemical pregnancies occurring at β-hCG values below 300. Although not statistically significant (*p*=0.091), multiple gestations exhibited a higher mean β-hCG level (2060.8) compared to single gestations (744.35). When β-hCG values were in the range of 500-1000, 45.5% of these corresponded to multiple gestations, whereas 40% of medians above 1000 were categorized as multiple gestations. The mean β-hCG for non-ongoing pregnancies (1166.9) was marginally lower than for ongoing pregnancies (1230.5) (*p*=0.922). Furthermore, the mean β-hCG was higher in cases of preterm births (1995.7) compared to term births (720.4), although this trend was not statistically significant (*p*=0.101). The mean β-hCG was also higher for transfers conducted on days 5-6 (3380.7) compared to days 2-3 (759.6) and when two embryos were transferred (1138) compared to one (730), yet these differences did not reach statistical significance.

Overall, a clinical pregnancy rate of 90.7% was achieved, with 24.1% categorized as multiple pregnancies (Table 1). The multiple pregnancy rates were observed to be 12.3% for women under 35 years, 25% for those aged 35-37 years, and 31.6% for women over 37 years (*p*=0.419). No significant correlation was observed between multiple pregnancy rates and maternal age; however, IVF techniques demonstrated a higher rate of multiple pregnancies (41.7%) compared to ICSI (10%). Of the cases categorized as multiple pregnancies, 76.9% utilized the IVF technique.

Among the 49 clinical pregnancies, 8 were excluded due to loss of follow-up, resulting in 41 cases available for analysis (Figure 1). Of the analyzed cases, 26.8% were classified as non-ongoing pregnancies and 73.2% as ongoing pregnancies (Table 2).

No significant correlation was found between maternal age and embryo evolution, with mean ages for ongoing pregnancies being 35.77 years and non-ongoing pregnancies being 36.27 years. In the IVF group, 70% of cases were ongoing, while in the ICSI group, 76.2% were ongoing; no significant correlation was established between the technique used and embryo evolution leading to live birth (*p*=0.655). However, IVF exhibited a higher rate of multiple live births (25%) compared to ICSI (14.3%). A majority (56.7%) of deliveries were via caesarean section (Table 2). No significant correlation was observed between maternal age and the mode of delivery. The caesarean section rate was 50% for IVF procedures and 62.5% for ICSI procedures (*p*=0.062).

In terms of gestational age, 40% of the births were classified as preterm (Table 2). The preterm birth rate was 50% in the IVF group and 31.3% in the ICSI group (*p*=0.296). Notably, singleton pregnancies had a preterm birth rate of 23.8%, while multiple pregnancies exhibited a significantly higher rate of 77.8% (*p*=0.006).

Analysis of neonatal outcomes revealed that 43.4% of firstborn infants and 62.5% of secondborn infants weighed less than 2500g (Table 2). Furthermore, in cases of multiple pregnancies, the average weight of the first newborn was 2198.33g, compared to 2892.14g for singleton pregnancies (*p*=0.019). No statistically significant relationships were detected between newborn weight and the technique employed, maternal age, day of transfer, β-hCG value, or type of embryo transfer.

**Figure 3:**
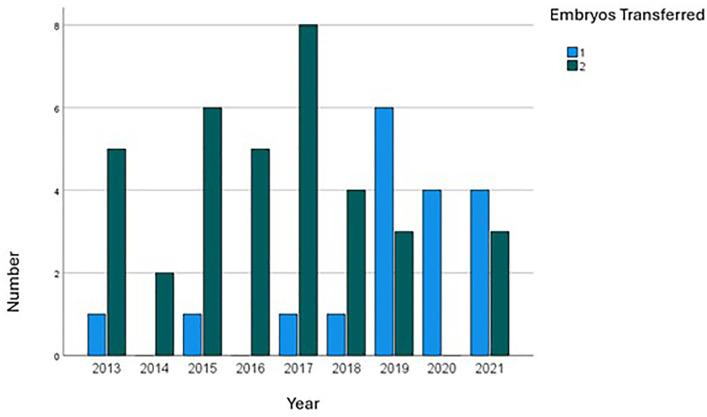
Trends in Embryo Transfer numbers over time in Oocyte Donation Cycles.

In the majority of procedures, two embryos were transferred (Table 3). However, a trend towards an increased number of eSET has emerged, particularly since 2019 (Figure 3). Across all age cohorts, the majority of embryo transfers involved the transfer of two embryos, with the highest incidence of eSET occurring among women aged 35 to 37 years. In IVF, two embryos were transferred in 91.7% of cases, compared to 46.7% in ICSI procedures. In instances where only one embryo was transferred, 88.9% of those were from ICSI (*p*<0.001). For the eSET cases, 5.6% resulted in multiple pregnancies; conversely, 33.3% of those where two embryos were transferred exhibited two embryos on ultrasound (*p*=0.079). Although not statistically significant (*p*=0.057), a higher ongoing pregnancy rate was noted for cases with two embryos transferred (82.1%) compared to those with a single embryo (53.8%).

The majority of embryo transfers occurred on days 2-3 (Table 3). There were no significant differences in the number of embryos visualized on ultrasound (singleton *versus* multiple pregnancies) when comparing transfers conducted at the cleavage stage to those performed at the blastocyst stage (*p*=0.569). Similarly, the rate of non-ongoing pregnancies was 27% for transfers on days 2-3, compared to 25% for blastocyst stage transfers (*p*=0.536). No significant relationships were found between the day of transfer and the occurrence of preterm births.

Embryo transfers using cryopreserved embryos (FET) accounted for 16.7% of cases, with maternal age being higher in this group compared to fresh cycles (*p*=0.116) (Table 3). The ongoing pregnancy rate following FET was 57.1%, compared to 76.5% for fresh cycles (*p*=0.293). Preterm births were observed in 75% of FET procedures and in 34.6% of fresh cycles. However, the majority of preterm births occurred in conjunction with fresh transfers (75%), although this did not reach statistical significance (*p*=0.125).

## DISCUSSION

### Sperm Donation

Sperm donation was initially exclusively utilized for the treatment of male infertility. However, in 2016, in Portugal, a regulation published in *Diário da República* announced that all women may access this treatment, regardless of their marital status, infertility diagnosis, or sexual orientation (Assembleia da República, 2017).

Currently, the indications for sperm donation have been extended to include female couples and women without a male partner. In the present study, the predominant indication for sperm donation was azoospermia, closely followed by cases involving combined male and female factors, and subsequently genetic causes. Notably, cases attributed to single-parent and transgender individuals constituted 2.2% of the total population studied.

The average age of participants in this study was 32.89 years, with the majority being under the age of 35. It was observed that second-line reproductive techniques were predominantly utilized among older women, specifically employed in 77.3% of cases involving participants over 37 years of age. This trend may be attributed to the decreasing success rates associated with first-line treatment modalities as maternal age increases, necessitating the use of more advanced interventions in older populations (Kim *et al.*, 2014).

In the present study, IUI is associated with a shorter duration of infertility as a first-line technique. This association is particularly relevant in cases of azoospermia and single-parent scenarios, as IUI involves less manipulation of gametes. In contrast, IVF and ICSI, classified as second-line techniques, are linked to a longer duration of infertility, secondary infertility, and situations where a female factor is implicated as a causal factor.

No statistically significant relationship was observed between the number of attempts and the progression of pregnancy, nor with other obstetric or perinatal complications. A study conducted in Israel in 2016 suggested that prior treatment involving sperm donation might reduce the risk of complications such as preterm birth or low birth weight, potentially due to changes in immune response (Fishel Bartal *et al.*, 2019). In this current study, no increased risk of complications was observed in the first attempt.

The first signal detected in maternal blood following implantation is β-hCG, which is commonly used as a diagnostic marker for pregnancy before ultrasound confirmation. Additionally, several studies suggest that it may serve as a predictive marker for embryonic development and for the occurrence of multiple gestations (Poikkeus *et al.*, 2002; Jayachandran *et al.*, 2012).

While the mean β-hCG levels trended higher in clinical pregnancies compared to biochemical pregnancies, the difference did not reach statistical significance. Most biochemical pregnancies exhibited β-hCG values below 100 mIU/mL, indicating the need for cautious interpretation of these findings. Furthermore, higher β-hCG levels were recorded in ongoing pregnancies, with 77.2% of non-viable pregnancies corresponding to values below 300 mIU/mL. The median β-hCG levels in multiple gestations were significantly greater than those observed in singleton pregnancies.

Regarding the risk of preterm birth, the literature fails to establish a correlation with β-hCG values, a finding that aligns with our results (Jayachandran *et al.*, 2012). It is also noteworthy that the mean β-hCG levels were elevated in cases where embryo transfer was conducted at the blastocyst stage, particularly with two embryos transferred; however, this potential confounding factor did not achieve statistical significance in our study.

Multiple gestation represents a significant concern associated with ART. Compared to the general population, ART procedures utilizing sperm donation demonstrate higher rates of multiple pregnancies, both in IUI and IVF, likely due to the use of hormonal treatments, ovulation induction, and the number of embryos transferred (Lansac & Royère, 2001). In the present study, the majority of pregnancies were classified as clinical, with 24.5% identified as multiple gestations. Although the higher occurrence of multiple pregnancies observed with second-line techniques was not statistically significant, it may be attributed to the fact that both IVF and ICSI involve embryo transfer, a topic that remains the subject of ongoing debate regarding the optimal number of embryos to transfer.

The predominant practice across all age groups was the transfer of two embryos; however, there is a notable trend toward a more equitable distribution over time, characterized by an increasing frequency of eSET. Importantly, when two embryos were transferred, the rate of ongoing pregnancies was higher, although this finding did not achieve statistical significance. Conversely, a significant association was established between the number of embryos transferred and multiple gestation, with a higher rate of multiple pregnancies occurring when two embryos were transferred.

Transferring two embryos has traditionally been common practice due to the increased likelihood of achieving at least one viable embryo and the corresponding boost in success rates of ART. Nonetheless, this practice is also linked to a greater incidence of multiple pregnancies, which can lead to a range of complications, including preterm birth, low birth weight, and a heightened rate of caesarean sections (Cutting, 2018; Wu *et al.*, 2020). The current study found a statistically significant relationship between multiple gestations and gestational age, with most multiple pregnancies resulting in preterm deliveries. Additionally, low birth weight was significantly linked to multiple gestations, as singleton pregnancies displayed notably higher average weights when analyzed separately from multiple pregnancies. However, no association was found between the mode of delivery and the type of pregnancy. In light of the increasing incidence of multiple pregnancies and the associated rise in complications, the adoption of eSET is currently being proposed as a strategy to improve neonatal outcomes (Tobias *et al.*, 2016).

The majority of pregnancies were found to be ongoing, with no significant differences noted in embryonic development across the various embryo transfer techniques. The incidence of multiple live births was higher with second-line techniques, consistent with the increased rates of multiple gestations.

A statistically significant relationship was identified between maternal age and the incidence of live births. Specifically, in cases where maternal age exceeded 37 years, most pregnancies were classified as non-ongoing, whereas approximately 80% of embryos developed successfully in women under 37 years old. These findings are consistent with existing evidence indicating that maternal age significantly impacts fertility, leading to a higher incidence of non-viable pregnancies in both natural conceptions and pregnancies achieved through ART (Cimadomo *et al.*, 2018). A study conducted between 2015 and 2019 compared the outcomes of ICSI cycles using donor sperm *versus* those using the patients’ own gametes. It found that in women over 37 years of age, the use of donor sperm is associated with a greater likelihood of successful embryonic development compared to cycles utilizing their own gametes. In this age group, rates of live births were higher, while rates of non-viable pregnancies were lower. Although advanced maternal age is generally considered a negative prognostic factor, the use of donor gametes may represent a more favorable option than treatment with one’s own gametes, especially in cases of significant male factor infertility (Mignini Renzini *et al.*, 2021).

The preterm birth rate was found to be 29.4%, while the average birth weight for singleton pregnancies was over 2500 grams, with low birth weight being more closely associated with multiple gestations. Evidence suggests that the use of donor sperm may be linked to a higher risk of complications, as the maternal immune system may not effectively adapt to the partner’s sperm, potentially increasing the risk of preeclampsia (Blazquez *et al.*, 2018). Consequently, this may be linked to other complications such as low birth weight or preterm birth (Yu *et al.*, 2018). However, studies are not conclusive regarding the relationship between these complications and sperm donation. Most studies do not find a significant difference when comparing donation and natural conception (Adams *et al.*, 2017). Similarly, when comparing IVF cycles with and without sperm donation, no differences were found in terms of the rate of live births/non-viable pregnancies, preterm birth, and birth weight (Gerkowicz *et al.*, 2018).

Although it is not a consistent association, some studies indicate that insemination with donor sperm is linked to a higher caesarean section rate. It is noteworthy that the control group consisted of pregnancies that did not use ART (Hoy *et al.*, 1999). In fact, ART is associated with an increased incidence of caesarean deliveries, which may serve as a confounding factor in the study. This increase is likely related to factors such as maternal age, pre-existing medical conditions, previous uterine surgery, and even obstetric complications (Stern *et al.*, 2018). In this study, caesarean delivery was indeed the most common type of birth, and second-line techniques were associated with a higher percentage of caesarean sections, although this was not statistically significant.

When comparing the developmental stage of embryos at the time of transfer, no significant differences were found regarding multiple pregnancies, live birth outcomes, or preterm delivery. While this was not observed in this population, it is important to highlight that blastocyst-stage transfer is a practice aimed at selecting embryos with the highest implantation potential. This approach is increasingly used to achieve a higher rate of live births, facilitate the transfer of a single embryo, and minimize the risks associated with multiple pregnancies and their complications. Although a higher rate of development is indeed observed, one would expect to see better neonatal outcomes. However, the literature indicates an increased risk of preterm birth and multiple pregnancies. Therefore, it is essential to consider both the long-term risks and benefits of transferring blastocyst-stage embryos *versus* cleaved embryos (Maheshwari *et al.*, 2016).

Fresh embryo transfer was associated with a statistically significant lower average maternal age compared to FET. FET is typically used following a failed fresh cycle, with remaining embryos, when endometrial response is inadequate during stimulation, or, for instance, when there is a risk of ovarian hyperstimulation syndrome (OHSS) necessitating a freeze-all strategy. Consequently, conception may occur later than expected, which accounts for the higher maternal age.

Several studies have indicated that FET may lead to better obstetric and neonatal outcomes, making it a viable alternative to fresh embryo transfer (Roque *et al.*, 2013). A meta-analysis published in 2017 evaluated 31 studies and concluded that FET is associated with a lower risk of low birth weight and neonatal mortality, with no significant association found with preterm birth. In fact, in this study, the rate of preterm birth was higher in FET, although no statistically significant relationship was observed concerning embryo progression, mode of delivery, or newborn weight (Sha *et al.*, 2018).

### Oocyte Donation

In this study, the average age of the recipients was 35.48 years, which is lower than the average reported by the Portuguese National Council for Assisted Reproduction in its most recent report, which was 41.8 years (Conselho Nacional de Procriação Medicamente Assistida, 2020). It should also be noted that it stipulates treatments are only eligible for public funding if performed before the age of 40, potentially narrowing the age range of the population at the Centro Materno Infantil do Norte Dr. Albino Aroso (CMIN).

Currently, there is a trend towards increasing maternal age, influenced by various social, occupational, and economic factors, which raises concerns regarding fertility. In the present study involving oocyte donation, the age of the recipient did not correlate with embryo development, multiple pregnancy rates, preterm birth, or birth weight. This finding is consistent with the literature, which suggests that age within this range is not associated with a lower likelihood of favourable obstetric outcomes (Kawwass *et al.*, 2013). It has also been suggested that implantation rates may be affected by the age of the donor rather than the recipient’s age. Given the careful selection of donors, oocyte donation represents a viable and increasingly utilized treatment option (Savasi *et al.*, 2016). However, it is documented that after the age of 45, there is a decline in obstetric outcomes, which is believed to be associated with paternal age as well (Yeh *et al.*, 2014) (Frattarelli *et al.*, 2008). Thus, oocyte donation may be considered a strategy to mitigate complications associated with advanced maternal age (Cimadomo *et al.*, 2018).

It is estimated that POI affects approximately 1% of women under the age of 40 and 5% of women between the ages of 40 and 45, representing a significant indication for oocyte donation (Golezar *et al.*, 2019). In the present study, the oocyte factor was the most common, accounting for 75.9% of the cases.

Although a statistically significant relationship was not found, it is understood that the underlying cause may influence the choice of technique utilized. In all instances of previous unsuccessful cycles, IVF was employed, while ICSI was used in cases with a concurrent male factor. In this study, the choice of technique was not influenced by the type or duration of infertility or the number of attempts.

As previously mentioned, the level of β-hCG is indicated as a predictor of ongoing pregnancy, as well as of multiple gestations (Metello *et al.*, 2016). In oocyte donation, the β-hCG level was higher in clinical pregnancies, whereas biochemical pregnancies consistently occurred below a threshold of 300. Multiple gestations, as well as ongoing pregnancies, exhibited higher average β-hCG values, although this did not reach statistical significance. The β-hCG level was also higher in instances of preterm births and when transferring blastocysts or two embryos; again, none of these findings were statistically significant.

A rate of 24.1% for multiple pregnancies was achieved, with the highest percentage associated with IVF, although this was not statistically significant. This technique was also linked to a greater proportion of deliveries involving more than one live newborn.

In cycles utilizing autologous gametes, it is common for the incidence of multiple gestations to decrease with advancing age; however, the use of donor oocytes may counteract this trend (Reynolds *et al.*, 2001). In fact, in this study, the rate of multiple gestations increased with advancing maternal age. Although this finding does not align with the typical expectations within the parameters of this investigation, it is noteworthy that a study conducted with women over the age of 43 found that oocyte donation was associated with a higher rate of multiple gestations and, consequently, an increased incidence of preterm births (Le Ray *et al.*, 2012). Additionally, it is common practice to transfer more than one embryo in older age groups to enhance the likelihood of achieving a pregnancy, which may further account for the observed rise in multiple gestations. This complication is prevalent and has significant obstetric and neonatal consequences, as previously highlighted; thus, promoting the eSET is particularly relevant, especially in advanced maternal age (Söderström-Anttila, 2001).

During the study period, an increase in eSET was observed, with this approach becoming the most prevalent method in the last three years. Nevertheless, for the overall population across all age groups, the majority of transfers still involved two embryos, primarily in the context of IVF. In this scenario, no statistically significant relationship was established; however, it was noted that both multiple gestations and ongoing pregnancies were more prevalent with the transfer of two embryos. Nonetheless, existing literature indicates that the consequences associated with multiple gestations should be a predominant consideration, necessitating a cautious approach to decision-making in this regard (Reynolds *et al.*, 2001; Clua *et al.*, 2012).

In addition to multiple gestations and their associated consequences, ART carries its own complications; however, there is a hypothesis that oocyte donation could be an independent risk factor for an increased rate of caesarean sections, low birth weight, and preterm deliveries. The literature on this topic is inconsistent, highlighting the importance of continuing to investigate these techniques and providing necessary care for expectant mothers (Shah *et al.*, 2019).

In the present study, 26.8% of pregnancies were classified as non-ongoing, with no influence from the technique employed. Although there are few studies in this area, there does not appear to be an increased risk of miscarriage associated with oocyte donation (Shah *et al.*, 2019; Jeve *et al.*, 2016).

Caesarean delivery rates are significantly elevated in ART. When compared to cycles using autologous oocytes, oocyte donation is associated with an increase in caesarean delivery rates. Indeed, in this study, more than half of the deliveries were via caesarean section, and this choice was not influenced by the technique used or the maternal age. It is believed that this may be related not only to the complications associated with ART but also to the anxiety inherent in the process (Jeve *et al.*, 2016; Shah *et al.*, 2019).

Regarding preterm delivery, studies exhibit controversy regarding its direct relationship with oocyte donation (Mascarenhas *et al.*, 2017). In this case, a rate of 40% for preterm deliveries was observed, which was not significantly altered by the technique employed. However, a relationship was found between preterm delivery and multiple gestations, with the incidence of preterm birth being much higher in multiple gestations, thereby reinforcing the importance of single embryo transfer.

Some studies have concluded that oocyte donation is associated with low birth weight (Jeve *et al.*, 2016; Shah *et al.*, 2019), however, the literature is not unanimous on this issue (Mascarenhas *et al.*, 2017), with this study reporting an average weight for the first newborn exceeding 2500g. Thus, low birth weight appeared to be more closely related to multiple gestations, with a statistically significant relationship established.

Obesity is considered a risk factor for non-ongoing pregnancies in ART. Although this relationship is controversial, studies indicate that it does not exist in the context of oocyte donation, as no association was observed between obesity and a higher rate of miscarriage or a lower rate of live births (Jungheim *et al.*, 2013). In the present study, no relationship was found between BMI and embryo development.

Embryos can be transferred at different developmental stages, making it essential to consider the risks and benefits associated with each stage. In oocyte donation cycles, most embryos were transferred at the cleavage stage; thus, it was not possible to establish valid statistical correlations regarding the embryo’s developmental state, as no association was observed with the number of embryos seen on ultrasound, ongoing pregnancies, or preterm births.

The type of embryo transfer (fresh *versus* FET) did not influence birth weight or the progression of the pregnancy; however, it is noteworthy that FET resulted in a lower percentage of ongoing pregnancies. Regarding preterm delivery, while the majority occurred after fresh transfers, the rate was higher in transfers involving cryopreserved embryos. A study conducted in 2020 considered a single-term pregnancy with a live newborn of appropriate weight for gestational age to be a good outcome, and it was found that in oocyte donation, fresh transfers had a higher likelihood of achieving this. This phenomenon is believed to be related to the lower risk of OHSS associated with oocyte donation, which can alter endometrial gene expression and increase the likelihood of complications (Roeca *et al.*, 2020). Nevertheless, considering the increasing use of cryopreservation and its documented better outcomes in cycles using autologous gametes, it remains crucial to continue studying its impact on oocyte recipients.

### Limitations and Strengths

One of the main limitations of this study is the absence of a control group. Pregnancies resulting from ART are known to carry a higher risk than spontaneous pregnancies, making it relevant to include a control group of women who conceived through ART using their own gametes. This study’s limited sample size for obstetric variables is also a significant limitation, potentially impacting the statistical power and generalizability of our findings. Future studies should aim to include larger cohorts and a control group representing spontaneous pregnancies.

Furthermore, the complications analysed in this study may be influenced not only by gamete donation but also by factors such as the ART procedure itself, the couple’s socioeconomic conditions, and other environmental and medical factors that may predispose individuals differently. Another limitation is that certain significant complications associated with gamete donation, such as hypertensive disorders, were not included in the analysis. Additionally, the potential interrelation among different complications further complicates the interpretation of results.

Despite these limitations, this study has notable strengths. The Centro Materno Infantil do Norte Dr. Albino Aroso (CMIN) serves as the headquarters of the *Banco Público de Gâmetas* (BPG), the only public gamete bank in Portugal, with two affiliated collection centers in Coimbra and Lisbon. Therefore, the data presented in this study accurately reflect national trends within the Portuguese National Health Service, providing a comprehensive and representative overview of gamete donation in the country.

## CONCLUSION

While this study observed trends indicating no clear association between gamete donation and outcomes such as preterm birth, low birth weight, or increased caesarean deliveries, these findings are limited by the retrospective design and lack of a control group. Thus, further research is essential to draw more definitive conclusions. However, it is important to note that multiple gestation is a significant concern in ART cycles, as it is itself associated with various complications. Promoting eSET particularly for older oocyte recipients is advisable.

Maternal age is indeed a factor that negatively influences the prognosis of these procedures, but gamete donation can still be a viable option with more benefits than using one’s own gametes, especially in the case of oocyte donation. The day of embryo transfer, the number of attempts, and the type of embryo transfer do not appear to be associated with better or worse neonatal outcomes. However, the value of β-hCG may be considered a predictive factor for clinical, ongoing, and multiple pregnancies.

Recipient couples should be informed and advised about the inherent risks of gamete donation, as well as the associated benefits and factors, to alleviate the anxiety associated with the process. Regular follow-up should be conducted to promote the well-being of the pregnant individual, the couple, and the newborn.
